# Multi-Service Highly Sensitive Rectifier for Enhanced RF Energy Scavenging

**DOI:** 10.1038/srep09655

**Published:** 2015-05-07

**Authors:** Negin Shariati, Wayne S. T. Rowe, James R. Scott, Kamran Ghorbani

**Affiliations:** 1 School of Electrical and Computer Engineering, RMIT University, Melbourne, VIC 3001, Australia

## Abstract

Due to the growing implications of energy costs and carbon footprints, the need to adopt inexpensive, green energy harvesting strategies are of paramount importance for the long-term conservation of the environment and the global economy. To address this, the feasibility of harvesting low power density ambient RF energy simultaneously from multiple sources is examined. A high efficiency multi-resonant rectifier is proposed, which operates at two frequency bands (478–496 and 852–869 MHz) and exhibits favorable impedance matching over a broad input power range (−40 to −10 dBm). Simulation and experimental results of input reflection coefficient and rectified output power are in excellent agreement, demonstrating the usefulness of this innovative low-power rectification technique. Measurement results indicate an effective efficiency of 54.3%, and an output DC voltage of 772.8 mV is achieved for a multi-tone input power of −10 dBm. Furthermore, the measured output DC power from harvesting RF energy from multiple services concurrently exhibits a 3.14 and 7.24 fold increase over single frequency rectification at 490 and 860 MHz respectively. Therefore, the proposed multi-service highly sensitive rectifier is a promising technique for providing a sustainable energy source for low power applications in urban environments.

AMBIENT energy harvesting is attracting widespread interest as it has the potential to provide a sustainable energy source for future growth and protection of the environment. Considerable research effort has been directed toward low-profile, low-power, energy efficient and self-sustainable devices aiming to harvest energy from inexhaustible sources such as solar energy, thermal, biomass, mechanical sources (e.g. wind, kinetic, vibration, and ocean waves) wastewater, and microwave energy. A thorough set of reviews is given in the literature^1^,^2^,^3^,^4^. Among these green energy sources, there has been a growing interest for radio frequency (RF) energy scavenging, as the availability of ambient RF energy has increased due to advancements in broadcasting and wireless communication systems. Furthermore, the development of wireless power transmission (WPT) technologies^5^ that allow micro sensors^6^, mobile electronic devices^7^, wireless implantable neural interfaces^8^ and far-field passive RFID (Radio-Frequency Identification) systems^9^,^10^,^11^ to operate without batteries has triggered impetus for RF energy harvesting.

Efficient RF energy harvesting is a very challenging issue, as it deals with the very low RF power levels available in the environment. Furthermore, the scavengeable power level can vary unpredictably, depending on several factors such as the distance from the power source, the transmission media, the telecommunication traffic density and the antenna orientation. The majority of available literature on RF rectification has been dedicated to narrowband rectennas, which essentially operate at a single frequency and hence provide low DC output power^12^,^13^. Various topologies, such as voltage doublers or multipliers have been employed in order to increase the RF to DC conversion efficiency and the output DC voltage for specific applications^14^,^15^,^16^. However, from an ambient RF scavenging perspective, harvesting energy from various available frequencies could maximize power collection and hence increase the output DC power. Ultra-wideband and broadband rectenna arrays have been proposed as a potential solution^17^,^18^. However in some cases, simulation and experimental results were not provided to demonstrate the findings^17^. A broadband rectenna consisting of a dual-circularly polarized spiral rectenna array operating over a frequency range of 2–18 GHz was demonstrated^18^. The rectified DC power was characterized as a function of DC load, RF frequency and polarization for power densities between 10^−5^ and 10^−1^ mW/cm^2^. However, the proposed rectenna was matched at a single input RF power level for a specified load resistance for the characterization. Also, due to the low Q value of the rectifier circuit, the conversion efficiency was a fraction of 1% at −15.5 dBm. From a design point of view, while it is relatively easy to achieve a broadband antenna, it is very challenging to realize a broadband rectenna due to the non-linearity of the rectifier impedance with input power across the frequency band^19^.

To address this, a promising approach is to use a dual-band or multi-band configuration. This can maximize the power conversion efficiency (PCE) at the specific frequencies where the maximum ambient signal level is available. Various dual-band RF energy harvesting systems has been demonstrated^20^,^21^,^22^,^23^,^24^, however a large signal analysis of the rectifier was commonly not provided over a broad input power range. A dual-band RF energy harvesting using frequency limited dual-band impedance matching has been proposed^20^ and the PCE was shown over a high power range of 0 to 160 mW, however it was only matched at a single input power level (10 dBm). A CMOS dual- narrowband energy harvester circuit was modeled at environmental power levels^21^. Again, the rectifier efficiency was demonstrated with only single input power levels of −19 and −19.3 dBm at 2 GHz and 900 MHz respectively, and a large signal analysis was not presented. A compact dual-band rectenna operating at 915 MHz and 2.45 GHz has been demonstrated^22^ and the PCE was shown for input power levels of −15, −9 and −3 dBm. However, the reflection coefficient was evaluated at a single incident power level. Furthermore, the efficiency results with dual-tone excitation simultaneously and single-tone excitation (at 915 MHz) are very similar, hence the impact of applying a dual-band technique does not demonstrate a clear advantage over a single band. A dual-frequency rectenna for WPT has been proposed^23^ which achieved a conversion efficiency of 84.4% and 82.7% at 2.45 and 5.8 GHz with a high input power level of 89.84 and 49.09 mW respectively. These power levels far exceed ambient levels in the environment^19^. A conformal hybrid solar and electromagnetic (EM) energy harvesting rectenna has been presented^24^ and the PCE was provided with −30 to 5 dBm input power, achieving an efficiency up to 40% at 1.85 GHz for higher input power levels (above −5 dBm). However the reflection coefficient was not provided at low input power range.

A multi-resonant rectenna that uses a multi-layer antenna and rectifier has been evaluated for a −16 dBm to +8 dBm RF received power level, but the rectifier circuit layout and large signal analysis were not provided to clarify the findings^25^. Furthermore, a rectenna for triple-band biotelemetry communications has been proposed using a triple-band antenna and single frequency rectifier^26^. However, this rectenna is not suitable for RF energy scavenging due to the low efficiency at lower input power levels. Another triple band rectenna presented an RF-DC efficiency over the input power range of −14 to +20 dBm^27^, however the reflection coefficient results were only evaluated at a single input power level. This rectenna was shown to harvest 7.06 μW of DC power from three sources simultaneously at a high input power level of +10 dBm. A multi-band harvesting system has also been proposed where four individual harvesters are designed to cover four frequency bands^28^. However, a large signal analysis was not provided over a broad input power range. Furthermore, the proposed harvesting system has a minimum sensitivity of −25 dBm, whilst in a real environment more sensitive systems are required as the available RF power levels are very low^19^.

Tunable impedance matching networks have been demonstrated in order to collect RF signals from various sources and convert them to DC power^29^. However from an application point of view, this is still single frequency rectification and it is not widely applicable to environmental RF energy scavenging where the available power is very low.

In order to increase the amount of RF energy scavenged by a rectenna, it is crucial to identify and harvest multiple ambient frequency sources over their realistic available energy range. Our previous research has demonstrated the feasibility of RF energy harvesting through RF field investigations and maximum available power analysis in metropolitan areas of Melbourne, Australia^19^. The maximum available power for different frequency bands based on antenna aperture and number of antennas in a given collection area was analyzed. Measured results and analysis indicated that cellular systems and broadcast sources are well suited to harvesting, with scavengeable RF power ranging from −40 to −10 dBm. This identifies two important considerations in the design of efficient rectenna for RF energy harvesting: the scavengeable ambient RF power sources available, and the significant variance of this power.

The RF to DC rectifier solutions proposed in recent literature have focused on maximizing the system efficiency at a given, and often quite high, input power level. This neglects the issues related to input power variation which can lead to unexpected variations in the matching network due to diode non-linearity. Also, the scavangeable levels of ambient RF power have been shown to be orders of magnitude lower. Therefore, based on our previous research outcomes and recommendations^19^, an efficient power harvesting solution could encompass a multi-band matching circuit at the specific frequencies where maximum signal power is available, enabling greater power harvesting due to the combination of RF signals. This also results in a higher power being fed to a single rectifier, utilizing the diode function more efficiently.

This paper presents an RF energy harvesting method that can scavenge a wide range of ambient power levels which are orders of magnitude lower than previous reported techniques in the literature. An efficient dual resonant rectifier circuit is proposed, matched to a 50 Ω input port at 490 and 860 MHz over a broad low input RF power range from −40 to −10 dBm. The proposed dual resonant matching network operates efficiently at two identified harvesting frequency bands over a wide input power range, maximizing DC power by scavenging two sources simultaneously.

The remainder of this paper is organized as follows. First, the key results for the reflection coefficient and output DC power are presented. Subsequently, the Discussion section summarizes the results and demonstrates their potential implications, the limitations of this study, open questions and future research. Finally, the Method section describes the proposed rectifier design.

## Results

**Figure 1:**
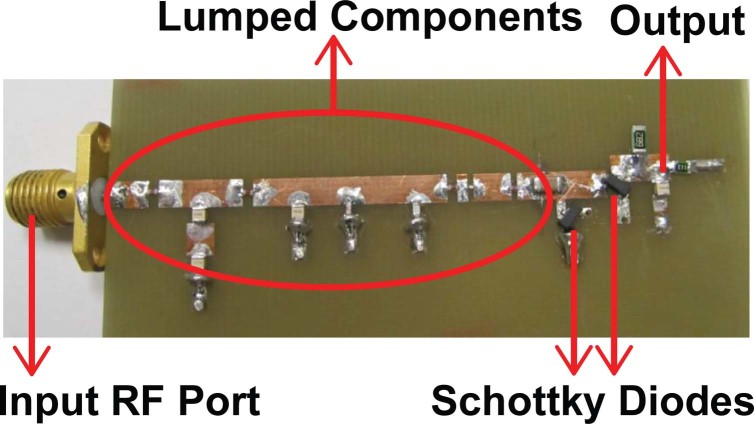
Fabricated rectifier prototype.

A dual resonant rectifier was fabricated on a 1.58 mm FR-4 substrate with a dielectric constant ε_r_ ≈ 4.5 and a loss tangent δ ≈ 0.025. These substrate parameters were measured using the Nicolson-Ross method^30^ so accurate values could be used in the rectifier design. A photograph of the fabricated dual resonant rectifier is shown in Fig. 1 which depicts input RF port, dual-band matching network lumped components, Schottky diodes and the output terminal. The performance of the rectifier was verified by measuring the input reflection properties, and the output power was calculated from the measured output DC voltage for the input powers from −40 to −10 dBm.

### Reflection Coefficient

**Figure 2:**
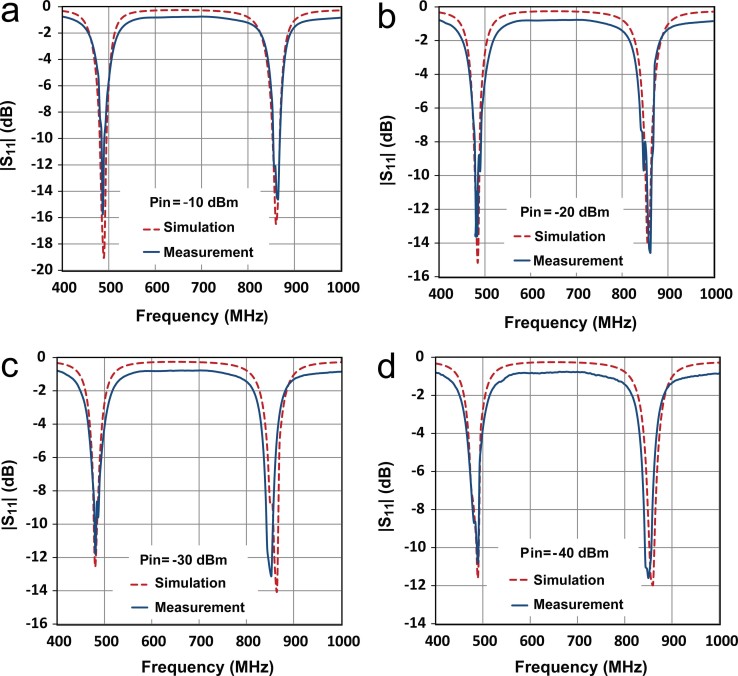
Simulated and measured |*S_11_*| as a function of frequency and input RF power for the proposed dual resonant rectifier circuit. (a) −10 dBm. (b) −20 dBm. (c) −30 dBm. (d) −40 dBm.

The |S*_11_*| of the rectifier was evaluated using a vector network analyzer (VNA). The VNA was re-calibrated for each input power level. Figure 2 compares the simulated and measured |S*_11_*| versus frequency for the dual resonant rectifier circuit at four different input power levels from −40 to −10 dBm. The measured results show very good agreement to the simulations. Slightly higher reflection was observed for the resonant frequencies at the lower part of the input power range (due to the diode characteristics). However, the proposed rectifier circuit is well-matched (|S*_11_*| < −10 dB) at the desired frequency bands of 478–496 MHz and 852–869 MHz over the broad range of input powers from −40 to −10 dBm. The small difference between simulation and measurement is due to the parasitic extraction accuracy.

### Output DC Power

In the frequency domain, the Harmonic Balance method of analysis provides a comprehensive treatment of a multispectral problem^18^. The method intrinsically takes into account the DC component and a specified number of harmonics, while allowing the ability to specify the source impedance and harmonic terminations. A Harmonic Balance simulation was used to numerically evaluate the output DC voltage of the dual resonant rectifier for both a single and two tone input. The output DC voltage across the load resistor was also measured and used to calculate output DC power. Measurements were performed using a Wiltron 68247B synthesized signal generator as a RF power source for the rectifier circuit. Recording of the output DC voltage across the load resistance was achieved with a Fluke 79III digital voltage meter. The RF source power was initially set at −10 dBm, and decreased in 2 dB steps. In the dual-band measurement case, two RF signal generators were fed to the rectifier circuit simultaneously via a power combiner.

**Figure 3:**
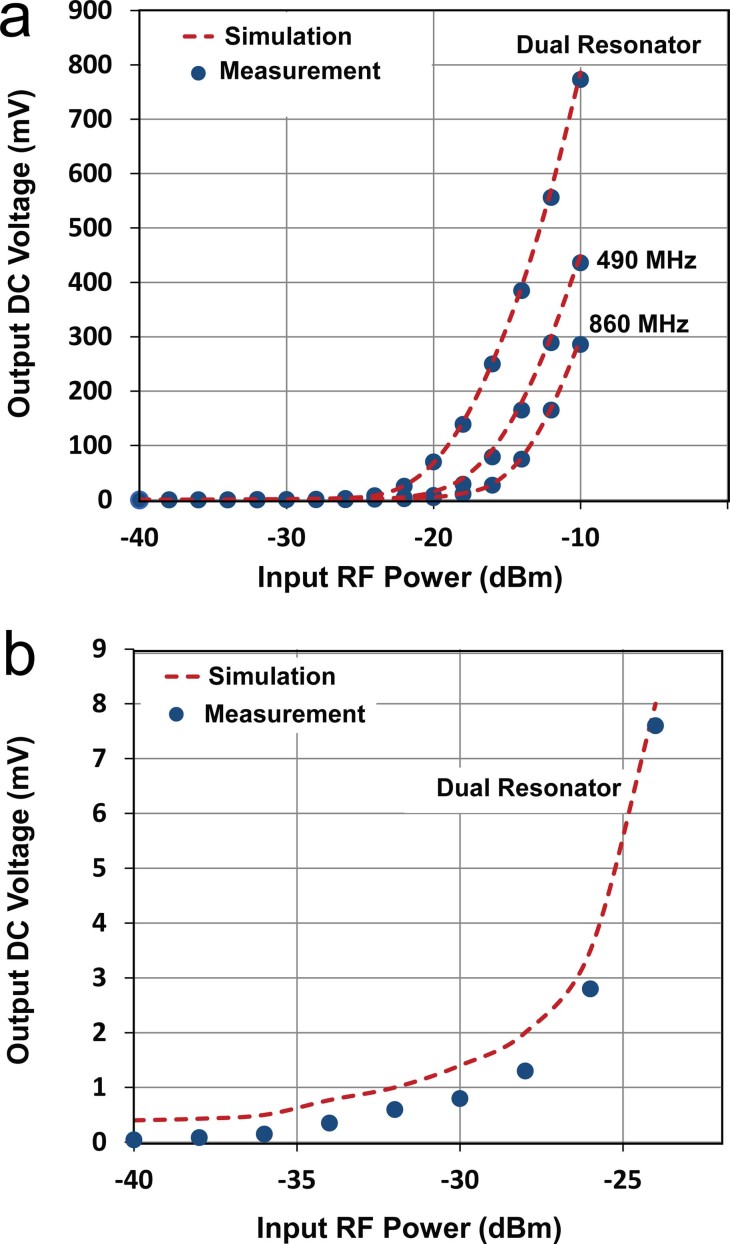
Output DC voltage as a function of input RF power for single input tone at both 490 MHz and 860 MHz and for dual input tones (a) with −40 to −10 dBm input RF power (b) with −40 to −25 dBm input RF power. (This power range is associated with the signal source).

The simulation and measurement results for single and dual input tones are summarized in Fig. 3(a) and (b). A measured DC voltage of 772.8 mV is achieved with two simultaneous input tones at an input power of −10 dBm. For single tone measurements, DC voltages of 436 mV and 286 mV at 490 MHz and 860 MHz respectively are produced. The comparison between the 490 and 860 MHz single rectifiers highlights the impact of the input frequency on the PCE. A higher amount of DC voltage can be generated at the lower frequency. This difference comes from decreasing diode performance at the higher frequency due to the higher junction capacitance of the diode^31^.

**Figure 4:**
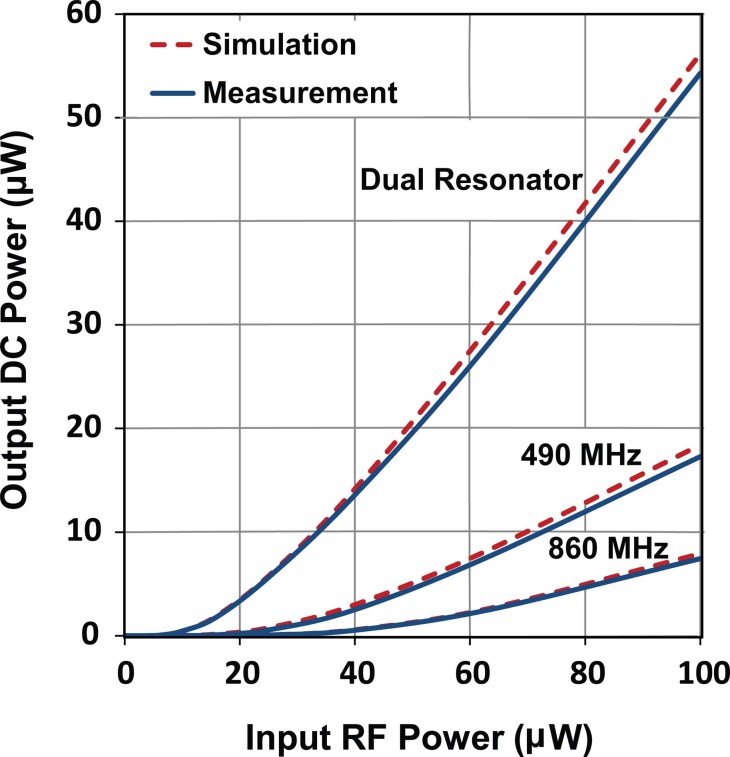
Output DC power as a function of input RF power for single and dual input tones.

**Figure 5:**
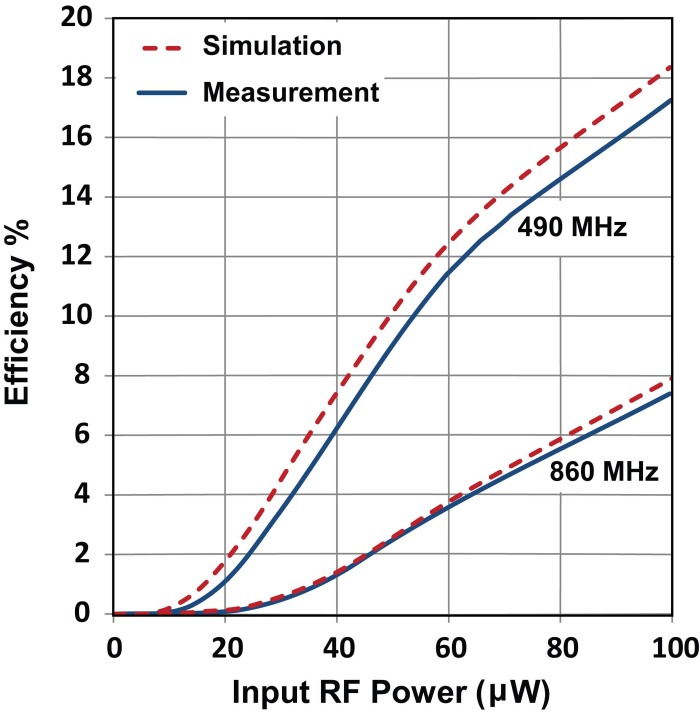
RF to DC conversion efficiency as a function of input RF power for single band rectification.

**Figure 6:**
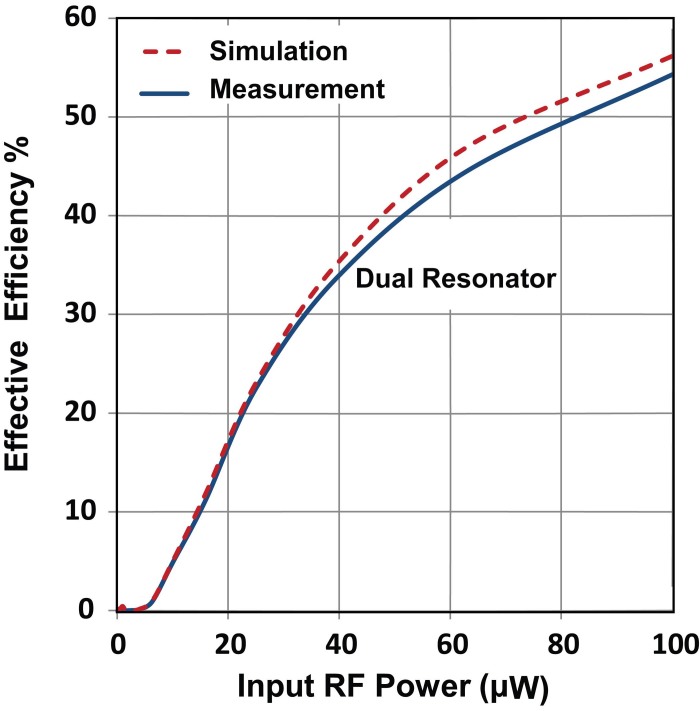
Effective RF to DC conversion efficiency as a function of input RF power for dual resonant rectification.

Importantly, a slightly higher DC voltage can be generated with the dual resonant rectifier as compared to the sum of output voltage from the two single bands, particularly at the lower input power levels as can be seen in Fig. 3(b) which shows the lower power section of Fig. 3(a) in more detail. By maximizing power collection from various sources of different frequencies and delivering the combined power to the rectification circuit, the diode conversion efficiency is enhanced which results in a higher level of rectified voltage. Figure 4 compares the simulated and measured output DC power for the dual resonant rectifier circuit with both single and dual input tones. A measured DC power of 17.3 μW and 7.5 μW can be generated at 490 MHz and 860 MHz respectively with a single tone input of −10 dBm (100 μW). This represents true efficiencies of 17.3% and 7.5% for the individual single band rectification (see Fig. 5). However, the measured DC output power with two concurrent input tones of −10 dBm is 54.3 μW which corresponds to an effective efficiency of 54.3% for the dual-band rectifier (see Fig. 6). This represents a 3.14 and 7.24 times increase in output DC power over the single tone excitation at 490 MHz or 860 MHz respectively. This trend is evident down to low input power levels (around 40 μW). Furthermore, there is a significant increase in the PCE of the dual resonant rectifier for lower input power levels (<40 μW).

Here, the effective efficiency is defined as the ratio of output DC power to the available input RF power rather than the power delivered to the diodes (equation (1)). The available power level is associated with the signal source. For the single resonator, the available input power is −10 dBm and the delivered power is also −10 dBm (assuming no loss). However, by creating a dual resonant matching network the power delivered to the diodes is −7 dBm (combined total input power from two signal generators) but the available power is still −10 dBm.
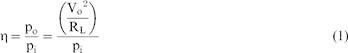


Therefore, combining input RF signals into a single rectification stage results in high sensitivity rectifier, which is widely applicable to real environmental RF energy scavenging. This multi-band technique can provide higher DC power than combining two separate single frequency rectifier circuits operating at the same frequencies. This is due to the fact that harvesting RF energy from various available sources simultaneously increases the delivered power to the rectifier, which improves the diode conversion efficiency and consequently enhances the output DC power. Table 1 summarizes this work as compared to previous published work.

In order to provide a realistic scenario for the proposed dual-band rectifier, measurement results were taken in three suburbs of Melbourne, Australia, congruent with our previous research outcomes^19^. Table 2 summarizes these environmental measurement results. It should be noted that the lower band (478–496 MHz) has a 3.67% fractional bandwidth and the higher band (852–869 MHz) has around 2% fractional bandwidth. Hence, various RF frequencies from different sources can be harvested within these two bands. The environmental measurement results demonstrate the feasibility of harvesting ambient EM energy from multiple sources simultaneously.

## Discussion

The feasibility of harvesting ambient EM energy from multiple sources simultaneously is investigated in this paper. The proposed dual resonant rectifier operates at two frequency bands (478–496 and 852–869 MHz), which are used for broadcasting and cellular systems respectively. The dual resonant rectifier exhibits favorable impedance matching over a broad input power range (−40 to −10 dBm) at these two bands. The achieved sensitivity and dynamic range demonstrate the usefulness of this innovative low input power rectification technique. Simulation and experimental results of input reflection coefficient and rectified output power are in excellent agreement. The measurement results demonstrate that a two tone input to the proposed dual-band RF energy harvesting system can generate 3.14 and 7.24 times more power than a single tone at 490 or 860 MHz respectively, resulting in a measured effective efficiency of 54.3% for a dual-tone input power of −10 dBm. It is evident that this dual resonant rectification technique increases the RF to DC effective conversion efficiency, and hence the recoverable DC power for low power applications. Furthermore from a design and economic perspective, utilizing a large number of components (e.g. antennas, diodes) to realize individual rectifier circuits for each frequency band creates additional expense. In order to provide more realistic measurement results, the proposed dual-band rectifier was tested in three suburbs of Melbourne, Australia. Therefore, this dual-band technique offers a simple and cost-effective solution which is of paramount importance for environmental power harvesting systems. This innovative technique has the potential to generate a viable perpetual energy source for low power applications in urban environments.

## Limitation of the study, open questions and future work

Utilizing diodes which are more suitable to low power applications (*P_i_* < −20 dBm) could increase the voltage sensitivity, resulting in a higher RF-DC conversion efficiency^32^. Applying a power optimized waveform excitation to the rectifier circuit in these frequency bands, a higher amount of DC power can be generated when compared with a single and dual tone excitations with the same input power^33^,^34^. However this technique is not applicable to energy harvesting where the input waveform is arbitrary.

Utilizing our proposed dual resonant rectification technique to combine resonant circuits for any other arbitrary frequency bands could lead to PCE improvement, provided that suitable diodes for the desired frequency bands are selected. Note that by increasing the operating frequency the rectification performance degrades due to the higher junction capacitance of the diode. Hence, lower output voltage is expected at higher frequency bands.

It is the object of our future work to design a multi-band rectenna array for enhanced RF energy harvesting. Furthermore, increasing the bandwidth, sensitivity and efficiency will also be investigated.

## Methods

The major goal in designing an efficient RF harvesting system is to produce high DC output power. Toward this goal, a high sensitivity rectifier is crucial for optimum RF scavenging. A significant factor governing the sensitivity of a rectifier is the threshold voltage of the diode used for rectification. The diode must be able to “switch on” for very low ambient energy levels.

**Figure 7:**
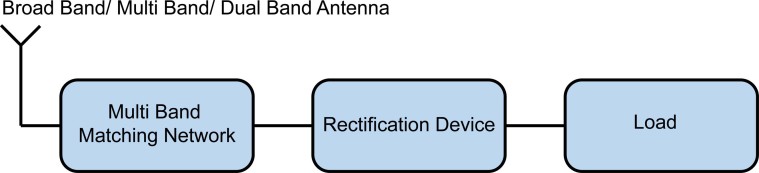
General block diagram of the RF energy harvesting system.

To address this sensitivity issue, a system that scavenges power from multiple frequency bands and combines them to activate a rectification circuit is proposed. The general block diagram of the proposed system is depicted in Fig. 7. Various environmental RF energy sources of different frequencies are collected by an appropriately designed antenna, and delivered to the rectification circuit via a multi-band matching network. The rectification circuit converts the combination of RF signals into DC power for low-power applications. The embodiment in this paper realizes a dual resonant matching circuit as a transition between a 50 Ω nominal antenna output and the non-linear rectification device at 490 and 860 MHz. Based on the Australian Radiofrequency Spectrum Plan^35^, these bands are allocated to broadcasting services and cellular systems.

### Device Selection

Due to the very low ambient power available in a real environment^19^, a very low threshold voltage rectification device is required in order to increase sensitivity. For this reason, Schottky diodes (GaAs or Si) are commonly employed for RF energy harvesting. In this work, a microwave Schottky detector HSMS2820 (*C_j0_* = 0.7 pF, *R_s_* = 6 Ω, I_s_ = 2.2e^−8^ A) is chosen due to its excellent high frequency performance, low series resistance (*R_s_*) and junction capacitance (*C_j_*), and low threshold voltage with high-saturation current^31^. This low threshold voltage (0.15–0.3 V) supports rectification at low input power levels.

### Proposed Rectifier Design

**Figure 8:**
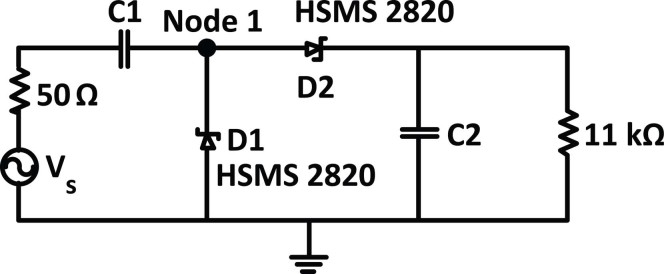
Schematic of a voltage-double rectifier without matching network.

In order to design an efficient RF harvesting system, the non-linearity of the rectifier impedance with frequency and input power should be matched to the 50 Ω output of the antenna at the desired frequency bands. Therefore, the diode input impedance as a function of frequency and different power levels were calculated and analyzed^36^. In order to match the input impedance of the rectifier to the 50 Ω output of the antenna, the total load impedance for different input power and frequencies should be determined. A circuit consisting of a pair of Schottky Barrier Diodes (SBD) terminated with a load resistor (*R_Load_* = 11 kΩ) and an output bypass capacitor (*C2* = 6.8 pF) was simulated using Agilent ADS software. Figure 8 shows the proposed geometry of the voltage-doubler topology^31^,^37^. The voltage doubler rectifier structure is employed for the design of the RF-DC power conversion system as this topology is well suited to low power rectification. The resistor and capacitor at the output will filter high frequencies. The high load resistor (11 kΩ) was chosen to observe a reasonable output voltage at very low currents. Using Large Signal S-Parameters analysis in Agilent ADS software, the load impedance and bypass capacitor were determined and optimized.

The voltage doubler rectifier in Fig. 8 consists of a peak rectifier formed by *D2* and bypass capacitor *C2* (6.8 pF) and a voltage clamp formed by *D1* and *C1* (total capacitance of the transmission lines and diode's parasitic capacitance (*C_p_*)). In the negative phase of the input, current flows through *D1* while *D2* is cutoff. The voltage across *D1* stays constant around its threshold voltage and the voltage at node 1 is charged to −*V_th1_* (where −*V_th1_* is the threshold voltage of the *D1*). At the negative peak, the voltage across *C1* is *V_amp_* −*V_th1_* (where *V_amp_* is the amplitude of the input signal). In the positive phase of the input, current flows through *D2* while *D1* is cutoff. The voltage across *C1* remains the same as the previous phase because it has no way to discharge. At the positive peak, the voltage across *D1* is 2*V_amp_* − *V_th1_*. Since *D2* is conducting current to charge *C2*, the voltage at the output is *V_out_* = 2*V_amp_* − *V_th1_* − *V_th2_*.

The DC equivalent circuit of the SBD is a voltage source in series with the junction resistor *R_j_* which is obtained by differentiating the diode voltage–current characteristic and is given by equation (2)^31^,^38^:
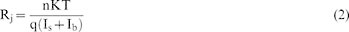


Where n is the diode ideality factor, *K* is the Boltzmann's constant, *T* is the temperature in degrees Kelvin, *q* is the electronic charge, *I_s_* is the diode saturation current and *I_b_* is the external bias current. At low power levels, the saturation current is very small (*I_s_* = 2.2 e^−8^ A) and for a zero-biased diode, *I_b_* = 0. Therefore, the resulting value of junction resistance at room temperature is approximately 1.7 MΩ. Since, the saturation current is highly temperature dependent, *R_j_* will be even higher at lower temperatures which tends to decrease the output voltage. As the input power increases, some circulating rectified current will cause a drop in the value of R_j_ and this phenomenon will increase the value of the DC output voltage. Furthermore, it is worth to highlight that the rectified current produced by the first diode (*D1*) in Fig. 8 constitutes the external bias current of the second diode (*D2*) which will help to reduce the *R_j_* and hence the detection sensitivity is improved. Therefore, depending on the amount of available bias current, *R_j_* is varying (equation (2)), hence the matching network is changing which impacts the amount of delivered power to the diode and results in different values of PCE.

**Figure 9:**
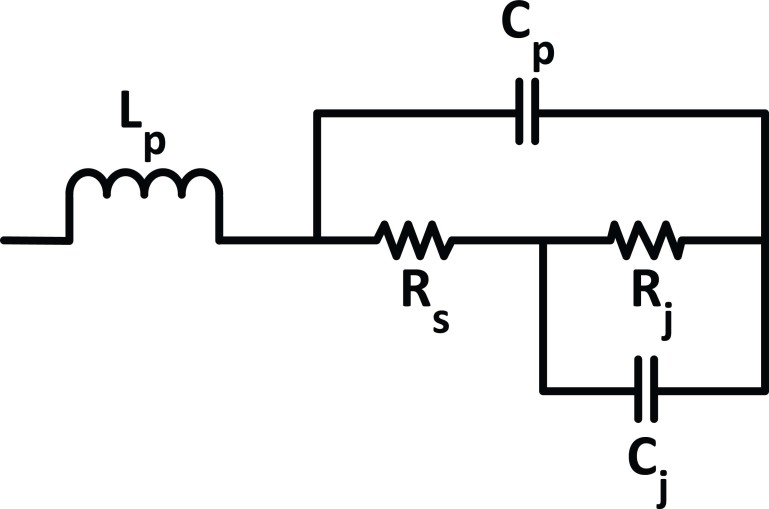
HSMS 2820 Schottky diode equivalent circuit.

A Schottky barrier diode can be modeled by the linear equivalent circuit shown in Fig. 9, where *L_p_* and *C_p_* are the diode's parasitic inductance and capacitance respectively due to packaging (*L_p_* = 2 nH and *C_p_* = 0.08 pF) which are generally unwanted^39^. This linear model is used for determining the diode impedance at a given input power.

**Figure 10:**
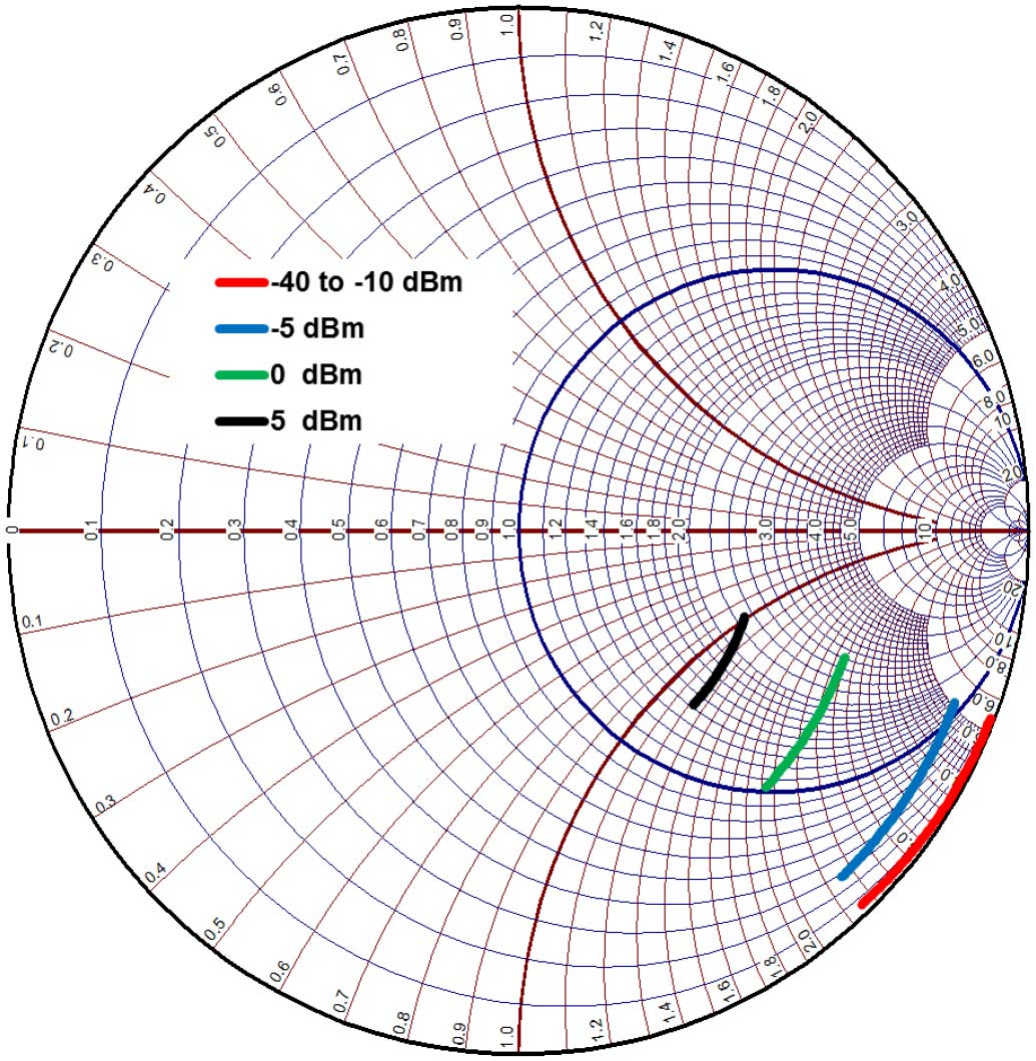
Diode input impedance calculated with Large Signal S-parameter analysis over the frequency range of 400 to 900 MHz with various unmatched input power levels.

The diode impedance analyzed using a Harmonic Balance simulator and a nonlinear model of the diodes over the frequency range of 400 to 900 MHz at various input power levels (Fig. 10). Due to the large junction resistor at low input RF power levels, the rectification device is turned off in absence of an appropriate matching network. Large Signal S-parameter analysis was conducted and higher input power (associated with the signal source) is applied directly to the Schottky diodes configuration of Fig. 8 which does not include a matching network in order to turn on the diodes (reduce the value of *R_j_*) and extract approximate input impedance value as our starting point in design of a matching network. As it can be seen in Fig. 10, with increasing the source power, the diode impedance is varying and it is beginning to switch on. Hence, the input impedance needs to be determined when the diode is turned on to realize the matching network for a rectifier circuit. Obviously, in the presence of an appropriate matching network the rectification device can be turned on at lower power levels, whilst in the absence of a matching network a higher input power should be applied to switch on the diode. (Note that, with an unmatched rectifier the total applied input power from the signal source cannot be delivered to the diode due to the high reflection in the circuit).

The aim is to match the input impedance of the device to 50 Ω at 478–496 MHz and 852–869 MHz bands over a broad range of input RF powers. The procedure commences by matching the diode input impedance at high unmatched source power and shifting the diode input impedance at various power levels to within the voltage standing wave ratio (VSWR) <2 circle on the Smith chart. This procedure assumes that diode input impedance does not drastically change in this low power range. The simulation results of Fig. 10 prove that this is the case.

**Figure 11:**
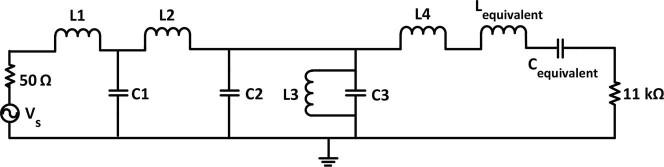
Schematic of a dual resonant rectifier (optimized parameters of the chip components are: *L1* = 3.9 nH, *C1* = 0.2 pF, *L2* = 12 nH, *C2* = 1.8 pF, *L3*′ = 3.9 nH, *C3*′ = 7.5 pF, *L4*′ = 11.6 nH, *L_equivalent_* ≅ 1 nH, *C_equivalent_* ≅ 1.3 pF).

In order to provide maximum power transfer from the antenna to the rectifier circuit, a dual resonant rectifier network is designed as a transition between a 50 Ω nominal antenna output and the non-linear rectification device over the power range of −40 to −10 dBm (see Fig. 11). Hence, a coupled-resonator structure with both series and shunt resonators is designed to achieve a dual-band network^40^. The linear equivalent circuit model of the SBD chip^39^ has been taken into consideration to design the dual band match at the desired frequency bands. In Fig. 11, *C_equivalent_* represents the total capacitance of the diodes and bypass capacitor and *L_equivalent_* is the overall parasitic inductance of the diodes. The series *L-C* resonator (*L4* + *L_equivalent_* and *C_equivalent_*) and the parallel L-C resonator (*C3* and *L3*) define the dual resonant circuit. The series resonator corresponds closely to the higher band specification of 852–869 MHz, whilst the parallel resonator approximates the lower 478–496 MHz band. A minimum number of components were used in order to reduce the ohmic and parasitic losses.

The resonant frequency of each sub-circuit was determined in isolation using the following equation:
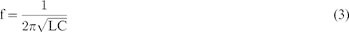


The 852–869 MHz band resonator circuit components were calculated. Here, *C* = *C_equivalent_* ≅ 1.3 pF consists of the combination of the bypass capacitor (6.8 pF) and the overall junction (*C_j0_* = 0.7 pF) and parasitic capacitance (*C_p_* = 0.08 pF) of *D1* and *D2*. Thus, *L* is calculated to be 26.5 nH in order to achieve an appropriate resonant frequency. Note that, *L* consists of *L_4_* and the overall parasitic inductance (*L_equivalent_* ≅ 1 nH) of *D1* and *D2*. The 478–496 MHz band resonator circuit components were calculated as *C3* ≅ 15 pF and *L3* ≅ 7.2 nH. Hence, the initial component values are determined for the two resonant circuits.

**Figure 12:**
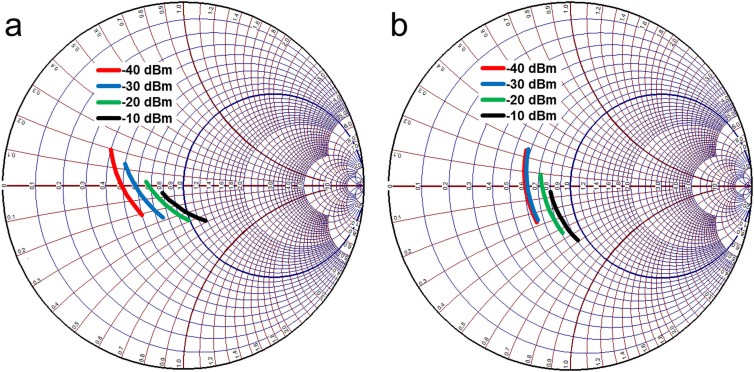
Dual resonant impedance matching with −40 to −10 dBm input RF power. (a) 478–496 MHz. (b) 852–869 MHz.

Initially these resonators were combined to achieve a dual-band structure. Then standard *LC* matching technique^40^ is utilized to determine *C1*, *C2*, *L1*, and *L2* to achieve minimum reflection at the resonant frequencies. The substitution of realistic chip component values with their associated parasitics, and addition of 50 Ω microstrip lines and T-junctions introduce delay and shift the imaginary part of the input impedance. The via-holes also contribute to extra inductance in the circuit. Hence minor circuit adjustments are made in order to fine tune the resonant frequencies to the desired values. The final optimized values of the standard chip components are: *L3*′ = 3.9 nH, C3′ = 7.5 pF and *L4*′ = 11.6 nH. Large Signal S-parameter analysis is also performed to demonstrate the matching network performance as the input power is varied. Simulation results for the input impedance of the circuit depicted in Fig. 11 are illustrated in Fig. 12. The proposed dual-resonant matching circuit achieves a VSWR <2 at 478–496 MHz and 852–869 MHz for input power ranging from −40 to −10 dBm. It should be noted that the matching circuit was designed based on the input impedance of two diodes and the output resistor and capacitor (Fig. 10). Therefore, selecting a different value for the load resistor requires a new matching circuit to be designed.

## Author Contributions

N.S. designed and simulated the rectifier, carried out the measurements, interpreted results and wrote the paper. K.G. directed the research, contributed to perform the simulation and measurement and validation of design and results. W.S.T.R. supervized the research, analyzed the data and contributed to the general concept, validation of design and results. J.R.S. analyzed the data and contributed to the general concept, validation of design and results. All authors reviewed the manuscript.

## Figures and Tables

**Table 1:** Rectifier performance comparison

Ref.	Technology	Measured Efficiency (%)	RF power variation (in PCE evaluation)	Rectification Technique
13	Schottky diode	82@50 mW	N/A	Single resonator
16	Schottky diode	44@−10 dBm	N/A	Single resonator
18	Schottky diode	20@0.07 mW/cm^2^	10^−5^ to10^−1^ mW/cm^2^	Broad band
		0.1@5x10^−5^ mW/cm^2^		
20	Schottky diode	77.13@22 dBm (158.49 mW)	0 to 160 mW	Dual resonator
21	CMOS	9.1@−19.3 dBm (900 MHz)	N/A	Dual resonator
		8.9@−19 dBm (2 GHz)		
22	Schottky diode	37 (915 MHz)@−9 dBm	−40 to 0 dBm	Dual resonator
		30 (2.45 MHz)@−9 dBm		
23	Schottky diode	84.4@89.84 mW (2.45 GHz)	0 to 100 mW	Dual resonator
		82.7@49.09 mW (5.8 GHz)		
27	Schottky diode	80@10 dBm (940 MHz)	−14 to 20 dBm	Triple resonator
		47@8 dBm (1.95 GHz)		
		43@16 dBm (2.44 GHz)		
29	Schottky diode	50@−5 dBm	−25 to 0 dBm	Dual resonator with tunable input response
This work	Schottky diode	54.3@−10 dBm	−40 to −10 dBm	Dual resonator
		11.25@−18 dBm		
		(490 and 860 MHz)		

**Table 2:** Environmental measurement results

Suburb	Available frequencies (MHz)	Respective available RF power (dBm) [μW]	Measured DC power (μW)
Bayswater	486, 488, 489, 490, 491, 867, 868, 869, 870, 871, 872, 873, 874	−19[12.5], −20[10], −17[19.95], −15[31.62], −22[6.3], −37[0.199], −37[0.199], −30[1], −24[3.98], −20[10], −30[1], −37[0.199], −40[0.1]	39.38
Bentleigh	491, 492, 494, 495, 865, 866, 867, 868, 869, 870, 871	−12[63.09], −46[0.02], −42[0.063], −57[0.001], −27[1.99], −27[1.99], −30[1], −37[0.199], −40[0.1], −40[0.1], −41[0.07]	30.9
RMIT University (Melbourne CBD)	487, 488, 489, 490, 491, 851, 861, 862, 866, 867, 868, 869	−30[1], −22[6.3], −29[1.25], −22[6.3], −20[10], −23[5.01], −21[7.94], −21[7.94], −30[1], −35[0.31], −40[0.1], −40[0.1]	14.5
